# Effect of periostin (OSF-2) on phagocytosis of *Plasmodium*-infected erythrocytes

**DOI:** 10.3389/fmicb.2025.1728562

**Published:** 2025-12-19

**Authors:** Joo-Yie Chin, Muhammed-Nur-Iman Mohammed-Syafiei, Yi-Jun Lim, Gordon Xue-Zhen Chong, Muhammad-Nasreen Suhaimi, Zhi-Ying Phong, Yee Ling Ng, Yee-Ling Lau, I-Ching Sam, Laurent Rénia, Wenn-Chyau Lee

**Affiliations:** 1 Department of Parasitology, Faculty of Medicine, Universiti Malaya, Kuala Lumpur, Malaysia; 2 Department of Medical Microbiology, Faculty of Medicine, Universiti Malaya, Kuala Lumpur, Malaysia; 3 Lee Kong Chian School of Medicine, Nanyang Technological University (NTU), Singapore, Singapore; 4 School of Biological Sciences, Nanyang Technological University (NTU), Singapore, Singapore; 5 A*STAR Infectious Diseases Labs (A*IDL), Agency for Science, Technology and Research (A*STAR), Singapore, Singapore

**Keywords:** OSF-2, phagocytosis, *Plasmodium falciparum*, *Plasmodium knowlesi*, rosetting

## Abstract

**Introduction:**

Phagocytosis is a pivotal component of the human innate immune defense against malaria. This essential defense mechanism is often modulated by various host-derived soluble factors. We investigated the phagocytosis of *Plasmodium falciparum*- and *P. knowlesi*-infected erythrocytes (IRBC) by human monocytic THP-1 cells in the presence of periostin (OSF-2), a human secretory protein involved in inflammation and tissue repair. This focus was prompted by the fact that OSF-2 is a potent stimulator of *Plasmodium* IRBC rosette formation, a parasite-derived cytoadherence phenomenon known to impede phagocytosis.

**Methods:**

Culturable parasite isolates were recruited, and tested with the THP-1 cells and recombinant human OSF-2 protein. The role of OSF-2 in IRBC phagocytosis by the phagocytes was evaluated in the presence and absence of uninfected erythrocytes (URBC), and the receptor involved was investigated with antibody blocking assay.

**Results:**

OSF-2 exerted a dual role. When rosetting was prevented via IRBC purification, OSF-2 increased IRBC phagocytosis. This stimulatory effect was also seen when THP-1 cells were primed with OSF-2 before IRBC exposure. This OSF-2-mediated phagocytosis was CD36-dependent and rapidly reversible upon OSF-2 removal. However, when rosetting was induced by the addition of URBC, the presence of OSF-2 reduced the rate of IRBC phagocytosis.

**Discussion:**

These findings highlight the complex parasite-host interactions influencing the infection pathogenesis.

## 1 Introduction

Malaria has imposed a significant healthcare burden on humanity. Despite numerous efforts and strategies by the World Health Organization (WHO) and various national malaria control programs across the world to eliminate it, nearly half of the global population remains at risk of contracting this parasitic infection (World Health Organization, 2024; Daily and Parikh, 2025). The situation is further complicated by the emergence of zoonotic malaria, as exemplified by the persistent reporting of symptomatic *Plasmodium knowlesi* infection in humans (Lee et al., 2022a). In 2023 alone, a total of 3,290 cases of knowlesi malaria were reported globally, with 14 deaths due to this zoonotic infection (World Health Organization, 2024). Several biological aspects of this simian malaria parasite differ from those of human malaria parasites (Cox-Singh et al., 2010; William et al., 2011; Braima et al., 2013; Lau et al., 2016; Liu et al., 2019; Lee et al., 2022b; Phang et al., 2023). These differences raise concerns about extrapolating knowledge gained from earlier human malaria studies to this potentially fatal zoonotic parasite. Nevertheless, the development of a stable *in vitro* cultivation system for *P. knowlesi* allows various pathobiological studies to be conducted more conveniently (Moon et al., 2013). This advancement mirrors the progress seen in falciparum malaria research following the establishment of continuous culture for *P. falciparum*, the most fatal human malaria parasite (Trager and Jensen, 1976).

Both *P. falciparum* and *P. knowlesi* induce architectural modifications to infected erythrocytes (IRBC) following invasion, albeit of different underlying mechanisms (Lee et al., 2019; Anderson et al., 2024). These substantial IRBC alterations increase their vulnerability to host immune defenses, particularly phagocytosis, a critical innate mechanism against *Plasmodium* parasites (Celada et al., 1983; Ayi et al., 2005; Lee et al., 2019; Alfaki and Elbasheir, 2025). While most research on *Plasmodium*-IRBC phagocytosis has concentrated on *P. falciparum* (Belachew, 2018; Chua et al., 2021; Musasia et al., 2022), the biological aspects of *P. knowlesi*-IRBC phagocytosis are not yet fully characterized. Furthermore, IRBC engulfment can be impeded by a parasite-derived phenomenon: rosette formation (Albrecht et al., 2020; Lee et al., 2020). Rosetting involves the adherence of an IRBC to several uninfected red blood cells (URBC), creating a stable, flower-like complex (Lee et al., 2022c).

We recently reported that the human-derived protein periostin (OSF-2)—whose expression is linked to inflammatory and pathological conditions—has a rosette-stimulatory effect (Phong et al., 2025). While the association between OSF-2 and phagocytosis has been established in cancer research (Kormann et al., 2020; Lin et al., 2022; Wei et al., 2023), its role in the phagocytosis of pathogens or pathogen-infected cells remains unknown. Therefore, in this study, we investigated the effect of OSF-2 on the phagocytosis of *P. falciparum*- and *P. knowlesi*-IRBC by human monocytic cells and evaluated the interplay between IRBC phagocytosis and rosetting in the presence of OSF-2.

## 2 Materials and methods

### 2.1 Materials used

Detailed information of materials used is available in Supplementary Table S1.

### 2.2 Study approval and general conditions of the experiments

The experiments were performed in full compliance with the guidelines established by both the Institutional Biosafety and Biosafety Committee (IBBC) of Universiti Malaya (UMIBBC/PA/R/FOM/PARA-025/2022) and the University of Malaya Medical Centre (UMMC) Medical Research Ethics Committee (MREC) (MRECID#2024312-13526). Eight biological replicates were used for all experiments in this study unless stated otherwise. For parasites, a biological replicate was defined as an individual parasite culture derived from a distinct culture batch, ensuring each contained unique infected red blood cells (IRBC).

### 2.3 Parasite and human monocytic THP-1 cell cultures

Laboratory-adapted *P. falciparum* and *P. knowlesi* (reference strains 3D7 and A1-H.1, respectively, unless stated otherwise) were thawed using the sodium chloride method and cultured at 2% hematocrit in RPMI 1640 media supplemented with AlbuMAX II and 10% (v/v) heat-inactivated human serum. Cultures were maintained under standard *in vitro* malaria parasite cultivation conditions, as previously described (Moon et al., 2013; Lee et al., 2014; Lee et al., 2022b; Phong et al., 2025). Late-stage parasite-IRBC were purified using a magnetic-activated cell sorting (MACS) system (Lee et al., 2021). Human monocytic THP-1 cells were cultured in fetal bovine serum (FBS)-enriched RPMI 1640 medium, under the standard *in vitro* cultivation conditions for mammalian cell lines, as described elsewhere (Lee et al., 2020).

### 2.4 Recombinant human periostin (OSF-2) protein solution preparation

Recombinant human periostin protein (hereafter, OSF-2) was reconstituted with 1X phosphate-buffered saline (PBS) (stock protein concentration: 100 μg/mL) according to the manufacturer’s manual (Phong et al., 2025). The stock was aliquoted and stored at 4 °C until use.

### 2.5 Phagocytosis assay

The MACS-purified late stage-IRBC (IRBC purity > 80%) were mixed with THP-1 cells in a physiologic ratio of 10,000 RBC: 1 monocyte, based on the normative values (derived from the routine complete blood count) of 5 million RBC/μl blood and 500 monocytes/μl blood in healthy adult individuals (El Brihi and Pathak, 2024). The cell suspension was exposed to different pathophysiology-relevant concentrations of OSF-2 (0, 100, 200, and 300 ng/mL) for 2 h under *in vitro* conditions (Zhu et al., 2016; Ding et al., 2018; Ninomiya et al., 2018; Phong et al., 2025). Phagocytosis was then assessed using the Giemsa-stained wet mount method (Lee et al., 2013). The IRBC phagocytosis rate was defined as the percentage of THP-1 cells that phagocytosed IRBC [ongoing (IRBC partially engulfed by the phagocytes)/successful (IRBC completely internalized, within the phagosome) engulfment of IRBC], determined by counting 1,000 THP-1 cells (Lee et al., 2020). To investigate how the duration of OSF-2 exposure affects IRBC phagocytosis, a separate experiment was performed. THP-1 cells were pre-exposed with 200 ng/mL OSF-2 for different time periods (0, 1, 2, and 4 h). Following this, the THP-1 cells were co-incubated with purified IRBCs for 2 h before the phagocytosis assay. In an additional experiment, six groups of THP-1 cells were primed with 200 ng/mL of OSF-2 for 2 h. Another aliquot of THP-1 cells “primed” with 1X PBS was used as the “OSF-2-free” control. Subsequently, THP-1 cells from the “OSF-2-free” group and one of the OSF-2-primed groups were incubated with purified IRBC for phagocytosis assay. For the remaining five groups of OSF-2-primed THP-1 cells, OSF-2 was removed from the suspension via centrifugation, washing, and re-suspension with culture medium. One washed group was immediately incubated with purified IRBCs for 2 h prior to the phagocytosis assay (as group “Hr_0_ post-OSF-2-removal”). This process was repeated for the remaining groups at different time points following OSF-2 removal (Hr_0.5_, Hr_1_, Hr_2_, and Hr_4_ post-OSF-2-removal) to observe the lasting effect of the priming.

The IRBC phagocytosis assay was repeated using human peripheral monocytes, with slight modifications. Briefly, the peripheral blood mononuclear cells (PBMC) were isolated using the Ficoll-Paque-based density gradient centrifugation method (Lee et al., 2020). The collected PBMC were incubated with CD14^+^ MicroBeads, followed by magnetic sorting using the LS columns. The purified CD14^+^ peripheral monocytes were divided into two groups, one of which was mixed with the purified *P. falciparum*-IRBC, whereas the other group was mixed with the *P. falciparum* parasite culture suspension. Each of these groups was further divided into two experiment settings, one was mixed with 200 ng/mL OSF-2 and the other served as the OSF-2 free control. The IRBC phagocytosis rates were determined following 2 h of incubation under the *in vitro* culture conditions. The experiment was repeated by replacing the peripheral monocytes with THP-1 cells.

### 2.6 Antibody blocking assay

To investigate the role of CD36 [a scavenger receptor essential for the non-opsonic phagocytic clearance of *Plasmodium*-IRBC (McGilvray et al., 2000)] in OSF-2-mediated IRBC phagocytosis, the IRBC phagocytosis assay was repeated with modifications, where one group was mixed with 10 μg/mL rabbit anti-human CD36 polyclonal IgG and OSF-2, another group was mixed with 10 μg/mL rabbit isotype IgG and OSF-2. The third group was mixed with OSF-2 alone without any antibodies, and an additional group without OSF-2 and any of the antibodies used was included as control. Subsequently, the mixtures of antibody and cell suspension were incubated for 2 h under *in vitro* conditions. This was followed by the phagocytosis assay to evaluate the antibody blocking effect of CD36 on the OSF-2-mediated IRBC phagocytosis.

### 2.7 Simultaneous evaluation of OSF-2-mediated rosetting and phagocytosis

THP-1 cells were added to the parasite culture suspension, which had a 2% parasitemia, at the previously mentioned RBC: THP-1 cellular ratio. The cell mixture was then divided into four groups. In the negative control, cells were treated with 1 × PBS. In the OSF-2 only treated group, cells were treated with 200 ng/mL OSF-2. In the positive control, cells were treated with 100 ng/mL insulin-like growth factor-binding protein 7 (IGFBP7), a reported rosette-stimulator that hampers IRBC phagocytosis (Lee et al., 2020). In the last group where a combined treatment with OSF-2 and IGFBP7 was applied, cells were treated with both 100 ng/mL IGFBP7 and 200 ng/mL OSF-2. Following a two-hour incubation, the phagocytosis and rosetting assays were conducted using the light microscopy-based Giemsa-stained wet mount method. Rosette is defined as a stable binding complex consisting of an IRBC with at least an uninfected erythrocyte (URBC). The rosetting rate was defined as the percentage of IRBC that form rosettes with URBC, determined by counting 200 IRBC under the immersion oil magnification (Lee and Rénia, 2020; Phong et al., 2025). The experiment was repeated using different laboratory-adapted *P. falciparum* strains and isolates (3D7, CS2, FVT402, FVT201, MKK183, WPP 3065, NHP4770, and NHP1106). The degree of OSF-2-mediated changes in IRBC phagocytosis and the degree of OSF-2-mediated changes in rosetting by the IRBC were determined.

### 2.8 Statistical analyses

GraphPad Prism 10.5 software was used for data analyses. Normality of the data was evaluated using the Shapiro–Wilk test. For paired comparison of two sets of normally distributed data, the paired *t*-test was used. For multi-group comparison based on one independent variable, One-way ANOVA with Tukey’s test was performed. For the comparison of multiple groups involving multiple independent variables, two-way ANOVA and three-way ANOVA were performed. Simple linear regression was performed to evaluate how the magnitude of rosette-stimulation by OSF-2 altered the IRBC phagocytosis. *p* values smaller than 0.05 were considered as statistically significant.

## 3 Results

### 3.1 Effect of OSF-2 on phagocytosis of purified IRBC by the human monocytic THP-1 cells

**Figure 1:**
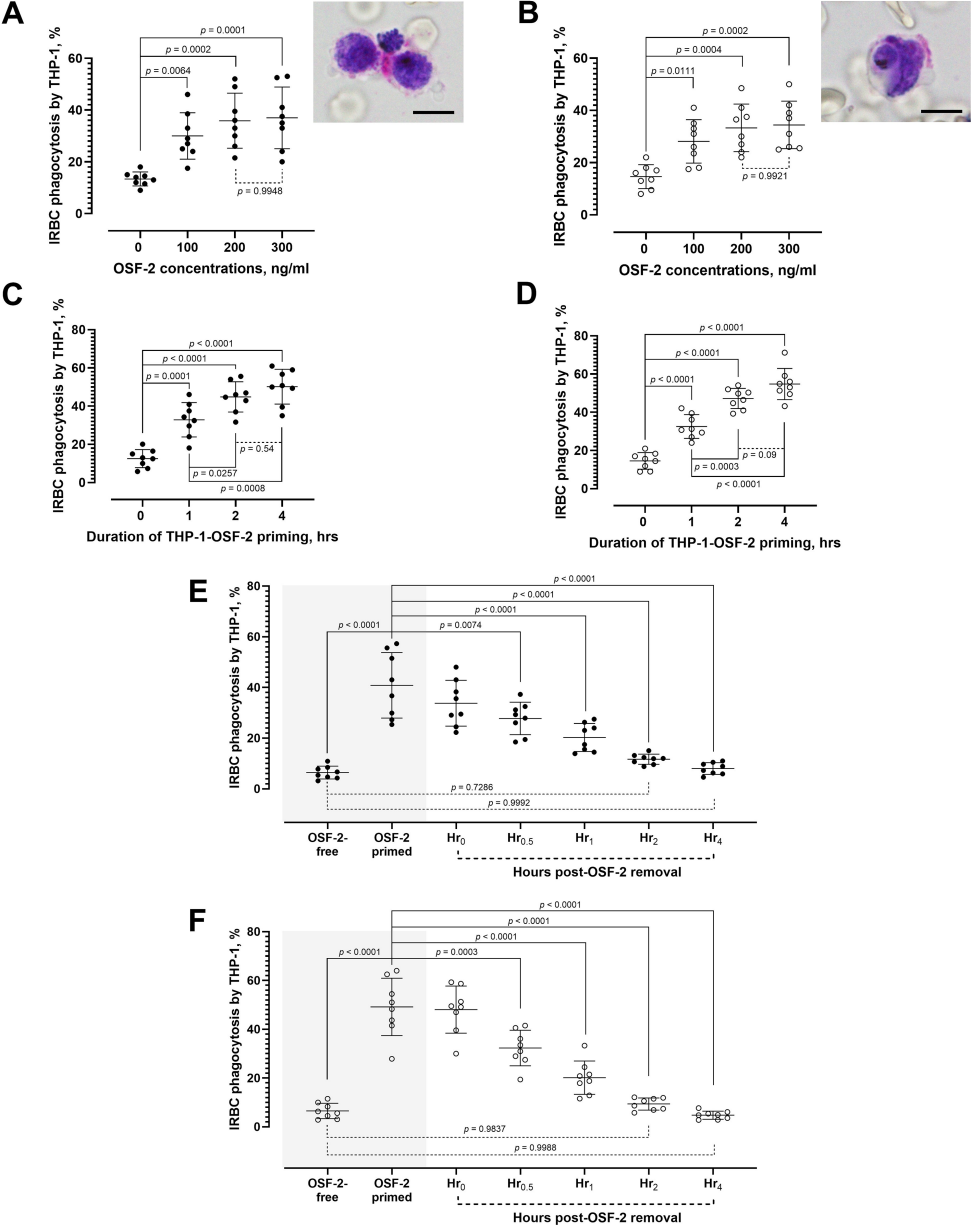
Characterization of OSF-2-mediated IRBC phagocytosis by the human monocytic THP-1 cells. Phagocytosis rates of purified IRBC by *P. falciparum* **(A)** and *P. knowlesi* **(B)** by THP-1 cells under different working concentrations of OSF-2, with inset photos showing an ongoing phagocytosis of a *P. falciparum*-IRBC (schizont) by two THP-1 cells, and a completely internalized *P. knowlesi*-IRBC (trophozoite) by a THP-1 cell; scale bar = 10 μm. Effect of different durations of OSF-2-THP-1 priming on the phagocytosis of purified IRBC by *P. falciparum* **(C)** and *P. knowlesi* **(D)**. Changes of phagocytosis rates by the human monocytic THP-1 against purified IRBC of *P. falciparum* **(E)** and *P. knowlesi* **(F)** after 2 h of priming THP-1 cells with OSF-2, followed by the monitoring of IRBC phagocytosis rates by the primed THP-1 cells at different times post-removal of OSF-2 from the culture system. One-way ANOVA was performed on all datasets. Error bars in the plots represent mean and S. D.

The THP-1 cells demonstrated similar baseline IRBC phagocytosis rates with *P. falciparum* (mean: 13.38%, S. D. 2.683) and *P. knowlesi* (mean: 14.63%, S. D. 4.588). The presence of OSF-2 significantly increased the phagocytosis rate of the THP-1 cells against the purified *P. falciparum*-IRBC (Figure 1A) and *P. knowlesi*-IRBC (Figure 1B) [one-way ANOVA *F*_3, 28_ = 11.10, *p* < 0.0001; and *F*_3, 28_ = 10.31, *p* < 0.0001, respectively]. For both species, IRBC phagocytosis rates by THP-1 cells increased with the OSF-2 concentrations, and plateaued at 200 ng/mL. In the subsequent experiments, the THP-1 cells were primed with 200 ng/mL OSF-2 for different durations, prior to the phagocytosis assay with purified IRBC. Priming with OSF-2 significantly enhanced the phagocytosis performance of THP-1 against *P. falciparum* (Figure 1C) and *P. knowlesi* (Figure 1D) [one-way ANOVA *F*_3, 28_ = 35.61, *p* < 0.0001; and *F*_3, 28_ = 66.35, *p* < 0.0001, respectively]. Notably, the IRBC phagocytosis performance improved with the duration of OSF-2 priming, where phagocytes primed for 2 h and 4 h demonstrated significantly higher phagocytosis rate than their counterparts that were only primed with OSF-2 for 1 h, in the case of *P. falciparum* and *P. knowlesi*. Nevertheless, no significant difference was found between the two-hour and four-hour priming for both *P. falciparum* and *P. knowlesi*. We also found out that the enhanced phagocytosis performance following OSF-2-priming was reversible. Upon removal of OSF-2 from the system, the rates of IRBC phagocytosis by the primed THP-1 cells reduced with the time post-OSF-2 removal toward similar levels of IRBC phagocytosis rates by THP-1 cells that were not primed with OSF-2 (one-way ANOVA *F*_6, 49_ = 29.81, *p* < 0.0001 for *P. falciparum*; and *F*_6, 49_ = 58.18, *p* < 0.0001 for *P. knowlesi*) (Figures 1E,F). For the experiments with purified *P. falciparum*-IRBC, significant reduction in IRBC phagocytosis rate by THP-1 cells (relative to that of the OSF-2-primed group) was found 30 min after the removal of OSF-2. The IRBC phagocytosis rates reduced further, where the IRBC phagocytosis rates recorded at two- and four-hours post-OSF-2 removal was of no significant difference from those by the non-OSF-2-primed THP-1 cells. Similarly for *P. knowlesi*, significantly lower IRBC phagocytosis rates (than that of the OSF-2-primed groups) were detected as soon as 30 min post-OSF-2 removal, which dropped further subsequently, toward the levels that were of no significant difference from that by the non-OSF-2-primed THP-1 cells. In summary, OSF-2 increased phagocytosis ability of THP-1 cells against the *Plasmodium*-IRBC, and the phagocytosis-boosting effect was reversible upon removal of OSF-2 from the system.

### 3.2 CD36 as the phagocytosis receptor involved in the OSF-2-mediated IRBC phagocytosis

**Figure 2:**
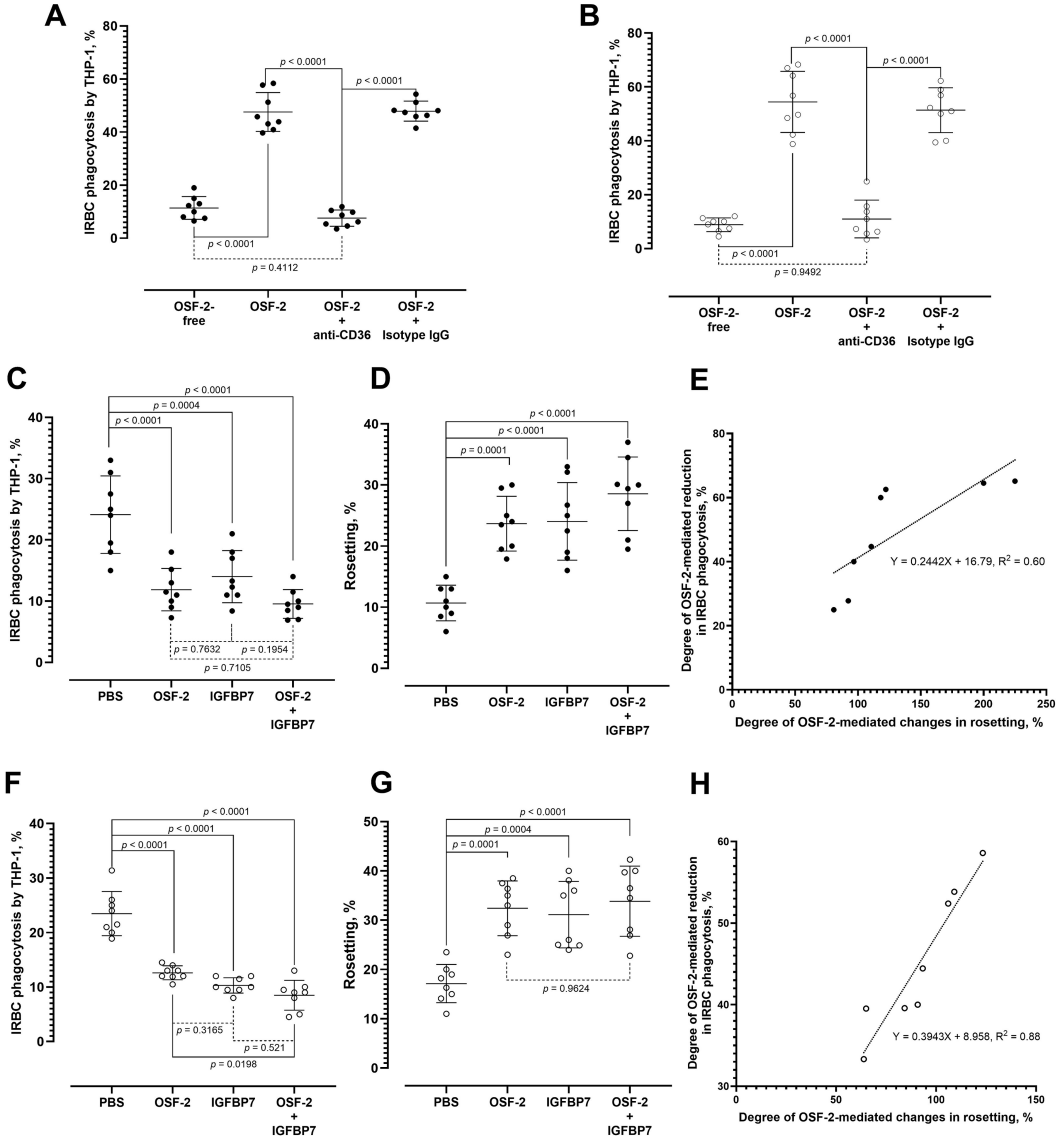
Dynamics of OSF-mediated IRBC phagocytosis and OSF-2-mediated rosetting by IRBC. Effect of antibody blocking of CD36 on the OSF-2-mediated phagocytosis of IRBC by *P. falciparum* **(A)** and *P. knowlesi* **(B)**. Culture suspension (containing URBC and IRBC) of *P. falciparum* was used to simultaneously evaluate the OSF-2 mediated IRBC phagocytosis by THP-1 cells **(C)** and OSF-2-mediated rosetting by the IRBC **(D)**. IGFBP7 was used as a positive control. Based on the data collected from **(C,D)**, linear regression revealed that the degree of OSF-2-mediated reduction in *P. falciparum*-IRBC phagocytosis followed the degree of OSF-2-mediated increment in rosetting (*R*^2^ = 0.6007; 95% CI: 0.045–0.443, *p* = 0.0239) **(E)**. Culture suspension (containing URBC and IRBC) of *P. knowlesi* was used to simultaneously evaluate the OSF-2 mediated IRBC phagocytosis by THP-1 cells **(F)** and OSF-2-mediated rosetting by the IRBC **(G)**. IGFBP7 was used as a positive control. Based on the data collected from **(F,G)**, linear regression showed that the degree of OSF-2-mediated reduction in *P. knowlesi*-IRBC phagocytosis intensified with the increment of OSF-2-mediated rosette-stimulation (*R*^2^ = 0.8825; 95% CI: 0.251–0.538, *p* = 0.0005) **(H)**. One-way ANOVA was performed on datasets **(A–D,F,G)**. Error bars in the plots represent mean and S. D.

The presence of anti-CD36 IgG significantly hampered the OSF-2-mediated phagocytosis of purified IRBC by *P. falciparum* (Figure 2A) and *P. knowlesi* (Figure 2B) (one-way ANOVA *F*_3, 28_ = 164, *p* < 0.0001; and *F*_3, 28_ = 78.10, *p* < 0.0001, respectively). Control experiments with the antibody isotype control did not display significant difference in the IRBC phagocytosis rates from the antibody-free, OSF-2-supplied group. There was also no significant difference between the OSF-2-free control and the group with OSF-2 and anti-CD36 antibodies.

### 3.3 OSF-2-mediated rosetting hampered phagocytosis of IRBC

We then assessed the impact of OSF-2 on IRBC phagocytosis by the THP-1 cells in a parasite culture suspension containing both IRBC and URBC, a condition that enables rosetting. This approach differs from the previously described experiments, which used purified IRBC, thus unable to form rosettes (Lee et al., 2019). IGFBP7 was used as a positive control, and we also evaluated the effect of co-exposure to OSF-2 and IGFBP7. In contrast to the earlier findings with purified IRBC, the rate of *P. falciparum*-IRBC phagocytosis significantly decreased when treated with OSF-2, IGFBP7, and the combination of both, compared to the negative control (Figure 2C) (one-way ANOVA *F*_3, 28_ = 17.45, *p* < 0.0001). However, the phagocytosis rates did not significantly differ among the OSF-2, IGFBP7, and OSF-2 + IGFBP7 groups. Concomitantly, the rosetting rates of the IRBC increased significantly with the presence of OSF-2 (Figure 2D) (one-way ANOVA *F*_3, 28_ = 18.05, *p* < 0.0001). No significant difference in rosetting rates was found among the experiment groups containing OSF-2, IGFBP7, and OSF-2 + IGFBP7. As the OSF-2-mediated rosette-stimulation increased, the degree of IRBC phagocytosis reduction intensified (Figure 2E).

With *P. knowlesi*, the rates of IRBC phagocytosis were also significantly reduced in the presence of either OSF-2, IGFBP7 and OSF-2 + IGFBP7, as compared to the negative controls (Figure 2F) (one-way ANOVA *F*_3, 28_ = 52.38, *p* < 0.0001). Interestingly, the *P. knowlesi*-IRBC phagocytosis rates from the group containing both OSF-2 and IGFBP7 were significantly lower than the setting containing OSF-2 alone. However, the IRBC phagocytosis rates between groups “IGFBP7” and “OSF-2 + IGFBP7” showed no significant difference. Simultaneously, *P. knowlesi* rosetting rates experienced significant increment in experiment groups containing OSF-2, IGFBP7, and OSF-2 + IGFBP7 (Figure 2G) (one-way ANOVA *F*_3, 28_ = 13.49, *p* < 0.0001). No significant difference in rosetting rates was found among the experiment groups containing OSF-2, IGFBP7, and OSF-2 + IGFBP7. With the increased OSF-2-mediated rosette-stimulation, the hampering of IRBC phagocytosis escalated (Figure 2H). In summary, IRBC in presence of OSF-2 formed more rosettes with URBC, leading to the reduced IRBC phagocytosis.

## 4 Discussion

**Figure 3:**
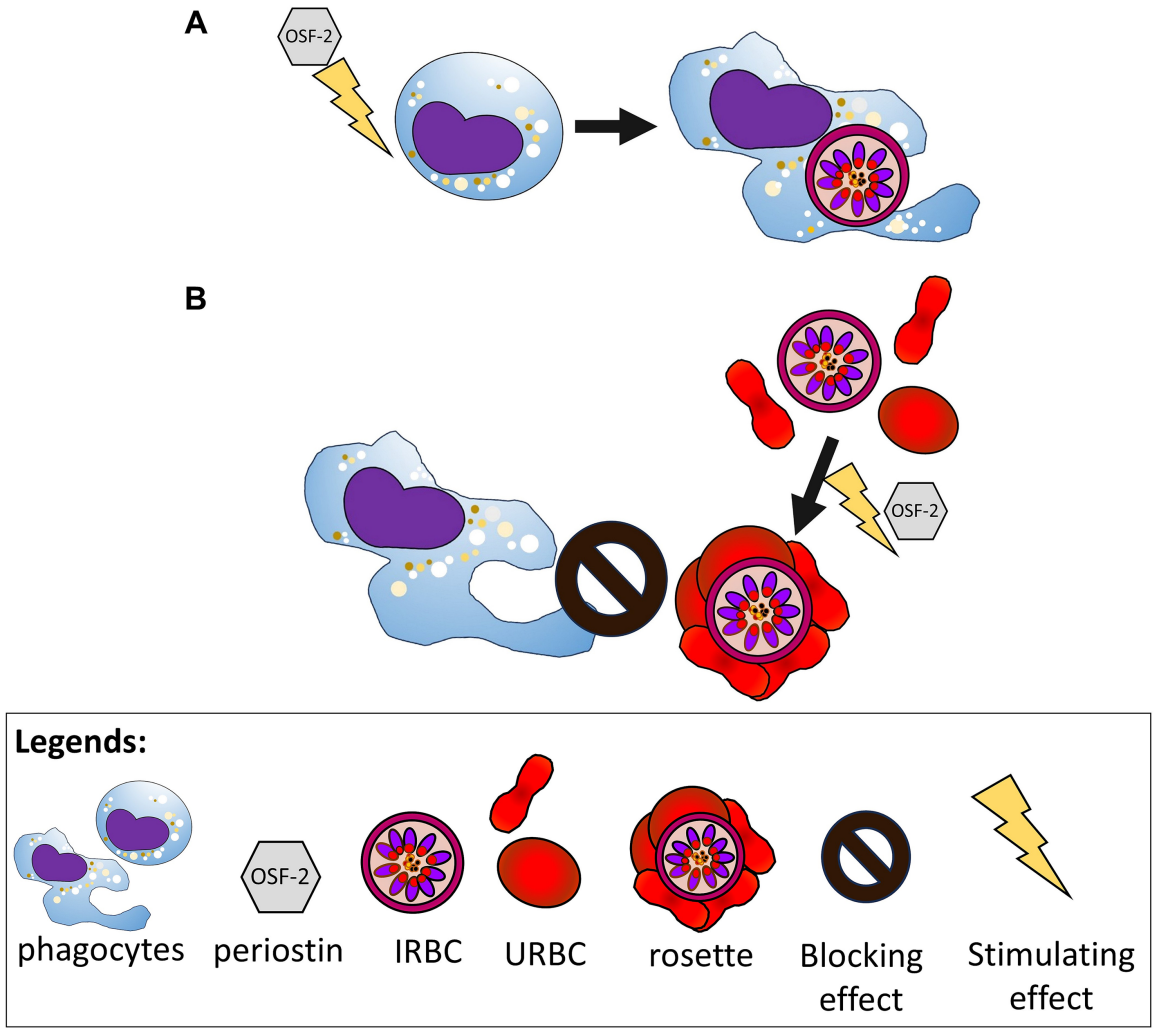
Schematic diagram illustrating the duo effects of OSF-2 on the phagocytes and *Plasmodium*-IRBC. **(A)** OSF-2 enhances the capability of phagocytes to engulf the IRBC. **(B)** Meanwhile the IRBC, upon exposure to OSF-2, are stimulated to form more rosettes, which hamper phagocytosis of IRBC by the phagocytes.

Phagocytic clearance of IRBC has long been acknowledged as an important innate immune response against malaria parasites (Celada et al., 1983; Ayi et al., 2005). The level of phagocytosis by host phagocytes can be influenced by various factors (Boero et al., 2023; Karavitis and Kovacs, 2011). Indeed, an inflammatory condition is one of the potent stimulators of phagocytosis (Aderem, 2003; Ishidome et al., 2017). Of note, OSF-2 is a secretory protein whose expression is increased by mesenchymal and immune cells during several inflammatory and pathological conditions (Liu et al., 2014; Li et al., 2015; Sonnenberg-Riethmacher et al., 2021; Phong et al., 2025). Interestingly, the reported role of OSF-2 in phagocytosis is context-dependent, often revolving around its inhibitory and modulatory effects (Kormann et al., 2020; Lin et al., 2022; Wei et al., 2023). In this study, we reported a new, context-dependent aspect of phagocytosis mediated by OSF-2 against malaria. When phagocytes were pre-exposed to OSF-2 and then presented with purified IRBC, the engulfment of IRBC increased significantly. Furthermore, the IRBC phagocytosis capacity improved with a longer OSF-2 priming period. However, the presence of OSF-2 in a mixture containing phagocytes, URBC and IRBC resulted in a lower IRBC phagocytosis compared to the OSF-2-free setting. This reduction coincided with a concomitant stimulation of the rosetting phenomenon by the IRBC. When IRBC are highly concentrated (for instance, during the sequestration of late stage-IRBC within the narrow microvasculature, leaving little room for IRBC to form rosettes with URBC), OSF-2 may facilitate the phagocytic clearance of the IRBC (Figure 3A). On the other hand, IRBC within larger vasculature will form rosettes in the presence of URBC and rosette-stimulators like OSF-2, thereby hampering IRBC phagocytosis (Figure 3B). Indeed, the extend of OSF-2-mediated rosette-stimulation could predict the degree of IRBC phagocytosis reduction, as observed in *P. falciparum* 3D7 strain and *P. knowlesi* A1-H.1 strain, as well as other laboratory-adapted *P. falciparum* strains and isolates (Supplementary Figure S1). It is important to note that the IRBC phagocytosis performance of human THP-1 cells was comparable to that of human peripheral monocytes (Supplementary Figure S2), allowing extrapolation of data from experiments with the THP-1 cells to the context of primary human peripheral monocytes in this study. To summarize, the role of human OSF-2 in phagocytic clearance of *Plasmodium*-IRBC depends on the availability of URBC, as the parasites use the URBC and OSF-2 as the building blocks for rosette formation (Phong et al., 2025), an immune-evasion strategy against phagocytosis.

An earlier study demonstrated that OSF-2 can promote the differentiation of macrophages with anti-inflammatory and tissue remodeling/ repair functions (Lin et al., 2022), and scavenger-like phagocytic function necessary for clearing apoptotic and defective cells (Woo et al., 2016). These scavenger phagocytes express high level of scavenging receptors, such as CD36 (Pennathur et al., 2015). Our study found that antibody blocking of CD36 significantly reduced the phagocytosis of purified IRBC, even in the presence of OSF-2. Our result demonstrates that CD36 is involved in the OSF-2-mediated phagocytosis of IRBC. The expression of CD36 on monocytes and macrophages is tightly regulated (Huh et al., 1996; Rac, 2025). Indeed, rapid translocation of intracellular CD36 to the cell surface of monocytes can be triggered by various pathophysiology-associated soluble factors (Yesner et al., 1996; Luiken et al., 2016; Wang and Li, 2019; Glatz et al., 2024). OSF-2 may serve as one of these signaling factors, leading to the enhanced phagocytic capacity of the cells. Of note, anti-CD36 antibodies reduced the IRBC phagocytosis rates to similar levels as the baseline IRBC phagocytosis rates, and did not completely inhibit IRBC phagocytosis. This reflects the complex array of receptors involved in the phagocytic clearance of *Plasmodium*-IRBC by human hosts (Chua et al., 2021; Alfaki and Elbasheir, 2025). In addition, the rapid reversal of the OSF-2-mediated phagocytosis enhancement upon removal of OSF-2 from the system suggests that OSF-2 may facilitate the binding interactions between the phagocyte surface CD36 and *Plasmodium*-IRBC, which is the crucial early step of non-opsonic phagocytic clearance of *Plasmodium*-IRBC (McGilvray et al., 2000). Alternatively, the rapid reversal of phagocytosis enhancement following OSF-2 removal may be due to a fast regulatory machinery controlling the phagocytic function. This may occur via the rapidly regulated surface expression of phagocytosis receptors by the phagocytes (Liao et al., 1994; Liao and Simon, 1994; Yesner et al., 1996; Zamora et al., 2012). Surface-expressed CD36, specifically, can be downregulated by monocytes and macrophages through internalization and proteolytic shedding (Liang et al., 2004; Luiken et al., 2016; Woo et al., 2016; Cai et al., 2022; Rac, 2025). Future studies, including those with more clinical isolates and samples of primary human monocytes, will be needed to investigate the precise mechanisms of OSF-2-mediated regulation of CD36 surface expression by the phagocytes. Besides CD36 that mediates non-opsonic phagocytosis, several other phagocyte receptors have been reported to be related to the phagocytic clearance of the malaria parasites. These include the Fc-gamma receptors (FcγR) and complement receptors (CR) that are involved in opsonization-dependent phagocytosis (Fernandez-Arias et al., 2013; Feng et al., 2021; Alfaki and Elbasheir, 2025), as well as other non-opsonic phagocyte receptors like several Toll-like receptors (TLR) that can recognize the parasite-derived nucleic acids, nucleic acid-associated products and malaria pigments (Gao et al., 2020; Köllisch et al., 2022; Alfaki and Elbasheir, 2025). Future studies may also look into the effect of OSF-2 on phagocytosis mediated by these receptors, which may be crucial for a better understanding on the biology of vaccine development against malaria parasites.

Interestingly, malaria parasites use OSF-2 as a trigger to form more rosettes that protect them from phagocytosis. Rosetting phenomenon has been demonstrated to act as a protective shield for the IRBC against phagocytes and other extracellular threats (Albrecht et al., 2020; Lee et al., 2020; Lee et al., 2021; Lee et al., 2022c; Lee et al., 2022d). Besides OSF-2, several other human-derived factors have been demonstrated to facilitate rosette formation by the *Plasmodium*-IRBC, as exemplified by IGFBP7 (Luginbühl et al., 2007; Lee et al., 2020). The rosette-stimulating factors may confer synergistic protection to the parasites against phagocytosis. Nevertheless, we observed significantly lower IRBC phagocytosis rates in the setting of OSF-2 + IGFBP7 co-exposure than the setting with sole OSF-2 exposure from experiments with *P. knowlesi* but not *P. falciparum*. Based on our studies, the environment that facilitated rosetting phenomenon hampered IRBC phagocytosis by approximately 60%. This effect may confer survival advantage to the parasites within the human host. Taken together, the findings from this study reflect the complex parasite–host interactions during pathogenesis, via series of strategies and counterstrategies derived from both parties.

## Data Availability

The raw data supporting the conclusions of this article will be made available by the authors, without undue reservation.
