# Supplementary material for: Effect of periostin (OSF-2) on phagocytosis of Plasmodium-infected erythrocytes
Source: Front Microbiol. 2025 Dec 19;16:1728562. doi: 10.3389/fmicb.2025.1728562 (PMC12757339; doi:10.3389/fmicb.2025.1728562)
Supplement: Supplementary file 1 [file Data_Sheet_1.pdf]

Supplementary Material

Supplementary table 1. Information on reagents and biological materials used in this study.

Reagent/Resource	Reference or source	Identifier/ Catalog Number / Notes
**Biological materials**		
Human monocytic THP-1 cell line	American Type Culture Collection (ATCC; ref code: TIB-202)	Used for all experiments
Parasite: A1-H.1 (*Plasmodium knowlesi*)	Laboratory-adapted parasite strain; maintained in Universiti Malaya.	Used for all experiments
Parasite: 3D7 (*Plasmodium falciparum*)	Laboratory-adapted parasite strain; maintained in Universiti Malaya and A*STAR ID Labs.	Used for all experiments
Parasite: CS2 (*P. falciparum*)	Laboratory-adapted parasite strain; maintained in Universiti Malaya and A*STAR ID Labs.	Used in experiments for supplementary figure 1
Parasite: laboratory-adapted *P. falciparum* isolates originated from Thailand:<br>FVT402<br>FVT201<br>MKK183<br>WPP3065<br>NHP4770<br>NHP1106	Laboratory-adapted parasite isolates; maintained in Universiti Malaya and A*STAR ID Labs; Lee et al.2020. doi: 10.7554/3Life.51546; Lee et al. 2021. doi: 10.1016/j.ebiom.2021.10368	Used in experiments for supplementary figure 1
**Chemicals, Enzymes and other reagents**		
1X phosphate buffer saline (PBS)	Gibco™	Cat#20012-027
AlbuMAX II™	Gibco™ ThermoFisher Scientific	Cat#11021-037
CD14 microbeads, human	Miltenvi Biotec	Cat#130-097-052
D-glucose	Sigma-Aldrich®	Cat#G7520-1KG
Dimethyl sulfoxide (DMSO)	Sigma-Aldrich®	Cat#D2650
Fetal Bovine Serum (FBS)	ScienCell™ Research Laboratories	Cat#0025
Ficoll-Paque solution	Cytiva	Cat#17144002
Giemsa	Sigma-Aldrich®	Cat#48900-500ML-F
Human IGFBP7 recombinant protein	R&D Systems	Cat#1334-B7
Hypoxanthine	Sigma-Aldrich®	Cat#H9377-25G
Immersion oil	Systerm	Cat#IM372-90
LD Columns	Miltenvi Biotec	Cat#130-042-091
LS Columns	Miltenyi Biotec	Cat#130-042-401
L-Glutamine	Sigma-Aldrich®	Cat#G8540-100G
Methanol	Friendemann Schmidt	Cat#M2097-4-4001
Periostin/OSF-2 (50µg)	R&D Systems	Cat#3548-F2
Rabbit anti-human CD36 polyclonal IgG	Sino Biological	10752-T24-100
Rabbit control isotype IgG	Sino Biological	CR1-10
RPMI 1640 medium	Gibco™	Cat#23400-013-1L
Sodium bicarbonate	Sigma-Aldrich®	Cat#S5761-500G
Sodium chloride	J.T.Baker	Cat#3624-69
Sodium hydroxide	Synerlab	Cat#SY-CS10237
Trypan blue	Sigma-Aldrich®	Cat#T6146
**Software**		
GraphPad Prism version 10.5	GraphPad	
**Others**		
0.5-10µl micropipette tips	Kirgen	Cat#KG5131-L
1.5ml microcentrifuge tube	GSBIO	Cat#CC102-N-F
1000µl micropipette filtered tips	Axygen	Cat#TF-1000-R-S
200µl micropipette filtered tips	Axygen	Cat#TF-200-L-R-S
25cm^2 cell culture flask canted neck (plug-seal cap)	SORFA	Cat#210110
3ml sterile transfer pipette	Biologix	Cat#30-0138A1
500ml vacuum bottle filter	Biofil	Cat#Fpe204500
50ml centrifuge tube	Biofil	Cat#CFT01115
50mL syringe	Terumo	Cat#SS*50LE
75cm^2 cell culture flask canted neck (plug-seal cap)	SORFA	Cat#210200
96-well microplates	Nunclon Surface	Cat#137101
Cellulose acetate syringe filter (0.22um pore size)	Bioflow Lifescience	Cat#MALCA25022
Cryovial	Corning	Cat#430488
Falcon® Cell Culture Flask T25, filter cap	VWR™	Cat#29185298

Glass slide	Sail brand	Cat#7101
Kimwipes	Kim Tech Science Brand	Cat#34155/34120
LD columns	Miltenyi Biotec	Cat#130-042-901
Microscope coverslip 22x22mm	Mariendfeld	Cat#0101052
Microscope slide with frosted side	Citoglas	Cat#P/N.0312-2101
Parafilm	Bemis	Cat#PM-996
QuadroMACS™ separator	Miltenyi Biotec	Cat#130-090-976
Vacutainer	BD	Cat#367284

Supplementary Figures

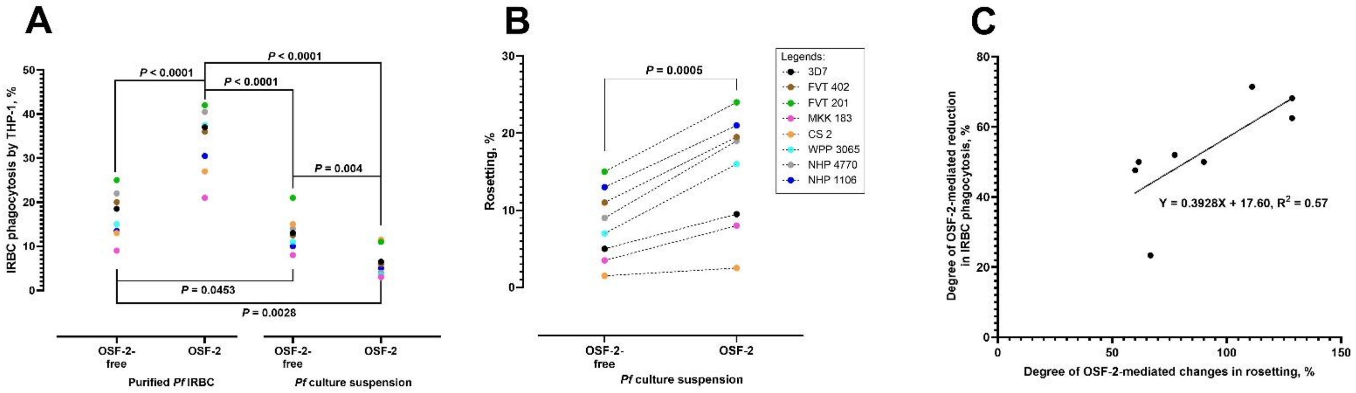

Supplementary Figure 1. Phagocytosis and rosetting assays repeated with different laboratory-adapted *P. falciparum* strains and isolates. (A) The IRBC phagocytosis evaluation with THP-1 cells was performed in two formats, i.e. with the purified IRBC (to prevent rosette formation) and culture suspension (rosettes formation was feasible). From the two-way ANOVA with Tukey's test, significance was found with regards to the availability of URBC ($F_{1,7} = 72.81$, $p < 0.0001$), and availability of OSF-2 in the system ($F_{1,7} = 108.7$, $p < 0.0001$). There was significant interaction between the URBC availability and OSF-2 availability ($F_{1,7} = 192.8$, $p < 0.0001$). Under the purified IRBC condition, OSF-2 increased the IRBC phagocytosis ($p < 0.0001$). However, the protein reduced the IRBC phagocytosis ($p = 0.004$) in the culture suspension. Under OSF-2 free condition, the IRBC phagocytosis recorded from experiment with culture suspension was lower than that of purified IRBC ($p = 0.0453$). Similar trend was found with the OSF-2 supplied setting ($p = 0.0028$). **(B)** Rosetting rates was simultaneously characterized during the phagocytosis assay with culture suspension. OSF-2 significantly increased the rosetting rates (paired t-test $p = 0.0005$, t = 6.161, df =7). Each parasite isolate was presented with different color, as indicated by the graph legend. **(C)** From the experiments conducted with the parasite culture suspension in **(A)** and **(B)**, linear regression revealed that the degree of OSF-2-mediated reduction in IRBC phagocytosis escalated with the increment of OSF-2-mediated rosette-stimulation ($R^2 = 0.5668$; 95% CI: 0.049 – 0.736, $p = 0.0311$).

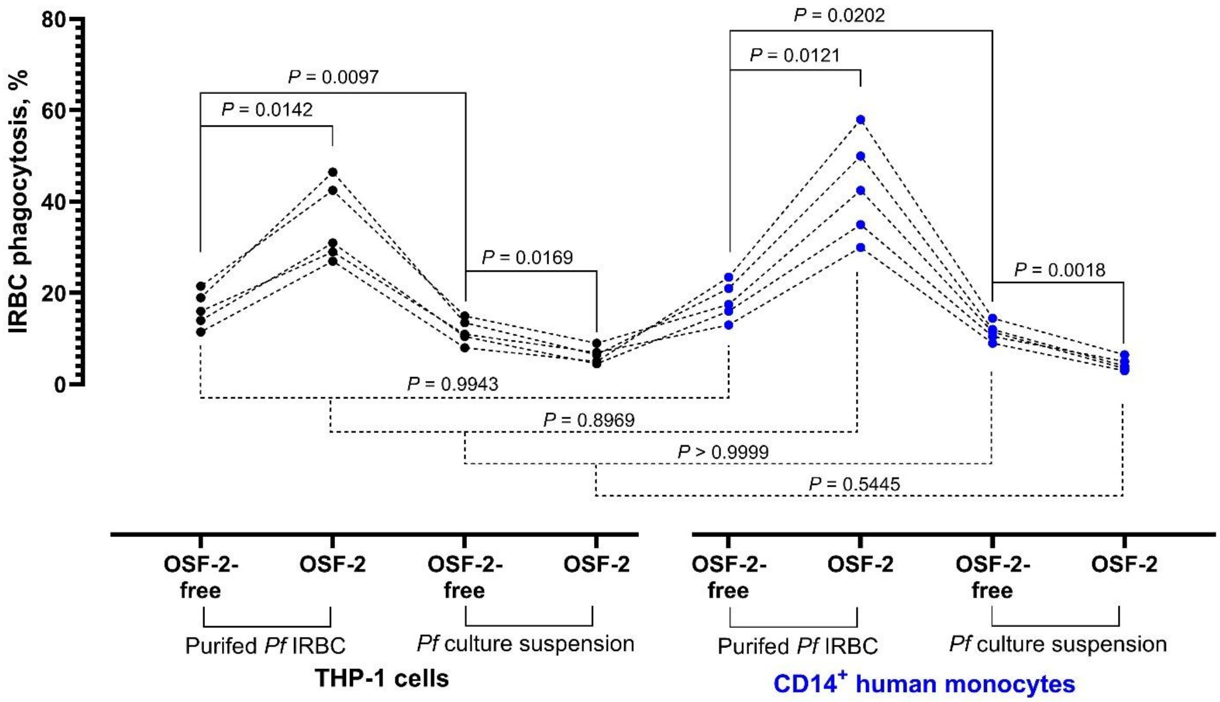

Supplementary Figure 2. Comparison of IRBC phagocytosis by the human monocytic THP-1 cells and the human peripheral CD14⁺ monocytes. The peripheral monocytes were derived from five healthy individuals. All experiments were conducted with *P. falciparum* 3D7 culture suspension (of different culture batches). Each experiment with the human peripheral monocytes was matched with experimentation with the THP-1 cells, as indicated by the dotted lines. From the three-way repeated measures ANOVA with Tukey's test, there was no significant difference between the THP-1 cells and $CD14^+$ human monocytes ($F_{1,8} = 0.4807$, $p = 0.5077$). Significance was detected with regards to the availability of URBC ($F_{1,8} = 134.2$, $p < 0.0001$) and the availability of OSF-2 ($F_{1,8} = 85.74$, $p < 0.0001$). From the experiments with purified IRBC, OSF-2 increased IRBC phagocytosis by THP-1 and human peripheral monocytes (p = 0.0142 and 0.0121, respectively); whereas experiments with culture suspension revealed that OSF-2 reduced the IRBC phagocytosis by THP-1 and peripheral monocytes (p = 0.0169 and 0.0018, respectively). Importantly, the IRBC phagocytosis rates by THP-1 and the peripheral monocytes were of insignificant difference when tested with OSF-2 using purified IRBC and parasite culture suspension (p = 0.8969 and 0.5445, respectively). Similar observation was made from experiments without OSF-2 ($p = 0.9943$ and $p > 0.9999$, for setting with purified IRBC and parasite culture suspension, respectively).
